# Odds and associated factors for thrombosis development among Lebanese COVID-19 patients: a case–control retrospective study

**DOI:** 10.1080/20523211.2024.2319743

**Published:** 2024-03-14

**Authors:** Mahmoud Youness, Sara Mansour, Fouad Sakr, Samer Olabi, Sarah Atwi, Iman Younes Martinez, Sami El Khatib, Souheil Hallit, Pascale Salameh, Diana Malaeb, Hassan Hosseini

**Affiliations:** a Research Department, Beirut Cardiac Institute, Beirut, Lebanon; b School of Pharmacy, Lebanese International University, Beirut, Lebanon; c Rafic Hariri University Hospital, Beirut, Lebanon; d Faculty of Medicine, Lebanese University, Beirut, Lebanon; e Department of Biomedical Sciences, Lebanese International University, Bekaa, Lebanon; f Center for Applied Mathematics and Bioinformatics (CAMB), Gulf University for Science and Technology, Mubarak Al-Abdullah, Kuwait; g School of Medicine and Medical Sciences, Holy Spirit University of Kaslik, Jounieh, Lebanon; h Applied Science Research Center, Applied Science Private University, Amman, Jordan; i INSPECT-LB: Institut National de Santé Publique, Epidémiologie Clinique et Toxicologie, Beirut, Lebanon; j Faculty of Pharmacy, Lebanese University, Hadath, Lebanon; k Department of Primary Care and Population Health, University of Nicosia Medical School, Nicosia, Cyprus; l School of Medicine, Lebanese American University, Byblos, Lebanon; m College of Pharmacy, Gulf Medical University, Ajman, United Arab Emirates; n Neurology Department, Henri Mondor Hospital, AP-HP, Creteil, France; o UPEC-University Paris-Est, Creteil, France; p RAMSAY SANTÉ, HPPE, Champigny sur Marne, France

**Keywords:** COVID-19, thromboembolism, factors associated, death, hospitalisation

## Abstract

**Background:**

Thromboembolism is reported to be up to 27% in COVID-19 patients due to SARS-CoV-2 infection. Dysregulated systemic inflammation and various patient traits play a vital role in thrombosis progression.

**Purpose:**

To assess odds and associated factors for thrombosis development among Lebanese COVID-19 patients.

**Methods:**

This was a case–control retrospective study conducted in January–May 2021. Patients infected with COVID-19 and developed thrombosis were classified as cases and patients who were thrombosis-free identified as control. A questionnaire assessed socio-demographics, clinical parameters, and WHO COVID-19 disease severity.

**Results:**

Among 267 patients, 26 (9.7%) developed thrombosis and the majority of thrombosis 34.6% was myocardial infarction, and the least (3.8%) was for catheter-related thrombosis. Results showed that the risk of thrombosis development is higher in patients with previous thromboembolic event (OR = 9.160) and previous intake of anti-hypertensive medications at home (OR = 3.116). However, females (OR = 0.330; CI: 0.118–0.925), intake of anticoagulants during hospital admission (OR = 0.126; CI: 0.053–0.300) and non-severe COVID-19 were at lower thrombosis risk (OR = 0.273). Patients who developed thromboembolic events had longer hospital stay (OR = 0.077).

**Conclusion:**

Patients with COVID-19 and thromboembolism were at higher risk of mortality as compared to patients with COVID-19 but without thromboembolism. The use of anticoagulants significantly reduced the risk for thromboembolism.

## 1. Introduction

Coronavirus disease 2019 (COVID-19), a global pandemic disease, is associated with severe respiratory illness, high intensive care unit (ICU) admission and mortality rates (Chen et al., 2020; Zhu et al., 2020). The overall mortality is estimated to vary between 2.3% and 12.8% (Johns Hopkins University Coronavirus Resource Center, 2020; Wu & McGoogan, 2020). Increased risk of venous thromboembolism (VTE), deep vein thrombosis (DVT), and pulmonary embolism (PE) are important complications reported in ICUs which suggest the need for thromboprophylaxis (Zhang et al., 2020). Previous literature recognised several reasons for the predisposition of thromboembolic events in COVID-19 patients (Ierardi et al., 2021). It is described that severe and critical cases are associated with systemic inflammatory response and hypercoagulable state (Zhang et al., 2020). Nevertheless, it is not thoroughly understood if COVID-19-associated haemostatic alterations are a direct cause of the virus, or the exclusive stimulation of the systemic inflammatory response syndrome and cytokine storm that contribute to thrombosis development (Borges et al., 2014; Mehta et al., 2020; Ramacciotti et al., 2019; Smither et al., 2019).

The mechanism of COVID-19 coagulopathy is related to dysregulation in the host response and massive release of cytokines which trigger a cascade of immune reactions inducing coagulopathy and thromboembolism (McGonagle et al., 2020; Qin et al., 2020). Interleukin-6 (IL-6) plays a key role in activating coagulopathy through increasing platelets and fibrin production, and provoking tissue factor expression (Kerr et al., 2001; Liu et al., 2020; Ranucci et al., 2020; Stouthard et al., 1996). The IL-6 levels are reported to be at least twice higher in COVID-19 patients on mechanical ventilation compared to community-acquired pneumonia patients on mechanical ventilation (Bacci et al., 2015; Herold et al., 2020; Ranucci et al., 2020). Another important mechanism that induces thromboembolism is related to hypoxia which is a common manifestation of severe COVID-19 that triggers thrombosis through the expression of transcription factors and thrombosis gene regulation (Gupta et al., 2019). In addition, fibrin accumulation in patients with severe acute respiratory syndrome coronavirus (SARS-CoV) is a consequent disturbance of plasmin and urokinase pathway balance (Gralinski et al., 2013). These processes contribute to massive microvascular thrombosis and thus diffuse pulmonary intravascular coagulopathy (Hadid et al., 2021).

COVID-19 manifested by systemic infection and inflammatory processes leads to coagulopathy development, increasing the need for intubation, and extending ICU stay (Zhang et al., 2020). Thromboembolic events are frequently observed by clinicians in hospitalised patients, nonetheless, there is anecdotal data on the true prevalence of thrombosis in COVID-19. Furthermore, most of the published international reports are collected from intensive care settings or enrolled by selecting high-risk or symptomatic patients (Ierardi et al., 2021). Growing evidence suggests that severe disease is linked to a pro-haemostatic condition, which may increase the risk of thromboembolism, although the data are still inconclusive (Al-Ani et al., 2020). A conspicuous elevation in the levels of D-dimer in COVID-19 patients is an important predictor of underlying coagulopathy and undesirable outcomes (Lippi & Favaloro, 2020). In fact, the disease can impact all coagulation parameters, though the degree of these changes and their relationship with illness severity and death are variable (Huang et al., 2020; Tang, Li, et al., 2020).

On 21 February 2020, Lebanon declared its first case of COVID-19 (Lebanese Ministry of Public Health, 2020). Immediate emergency measures were implemented by providing the necessary medical human resources and allocating specific departments and ICU sections for COVID-19 patients (Kerbage et al., 2021). Conversely, the public health and hospital sectors continue to suffer due to the severe economic crisis that is hitting the country for the last 2 years with a concurrent state of political corruption and financial collapse (Bizri et al., 2021). One published report assessed the relationship between COVID-19 and thrombosis, but the prevalence and risk factors of development have never been comprehensively investigated although it is highly associated with mortality and morbidity including acute limb ischemia, myocardial infarction (MI), acute coronary syndrome, venous thromboembolism, acute cerebrovascular accident, and disseminated intravascular coagulation (Avila et al., 2021). Thrombosis development is considered a complication in COVID-19 patients whereby identifying the specific associated risk factors and highlighting the potential treatment options will provide an impact not only on local level but also on a global aspect. Therefore, this study aims to fill the gap in knowledge about the prevalence and the factors associated with thrombosis development since thromboembolic events represent a significant health risks for hospitalised patients. Furthermore, thrombosis treatment in COVID-19 patients poses substantial challenges to healthcare providers due to morbidity and mortality and the availability of various treatment strategies. Thus this study aims to investigate the odds of thromboembolic events and the associated factors for thrombosis development in hospitalised Lebanese patients.

## 2. Materials and methods

### 2.1. Study design and participants

A case–control retrospective study was conducted between January and May 2021 at four Lebanese university tertiary hospitals. All hospitalised patients during the period of the study and diagnosed with COVID-19 by reverse transcriptase-polymerase chain reaction (RT-PCR) assays performed on nasopharyngeal swab specimens were considered eligible for study enrolment. Cases were defined as patients with COVID-19 infection who developed thrombosis during hospitalisation, confirmed by diagnostic procedures such as a Computed Tomography (CT), Magnetic Resonance Imaging (MRI) of the brain, Magnetic Resonance Angiography (MRA), electrocardiogram, echocardiography, echo Doppler, laboratory tests (such as troponin), and accompanied by clinical signs and symptoms. The thromboembolic events were confirmed by physician diagnosis in the charts and included blood clots in the deep veins of the legs, blood clot in the lungs, blood clot in the brain, or blood clot in the heart.

Control patients were identified from inpatients with a confirmed diagnosis of COVID-19 infection without evidence of thrombosis development during the study period. We excluded patients with factors that increase the propensity for the development if thrombosis as pregnancy, cancer.

### 2.2. Data collection

Data were collected using a standardised data collection form written in English. The data collection sheet was developed to assess the different factors associated with thrombosis development based on other studies (Ierardi et al., 2021; Lippi & Favaloro, 2020) and validated through a pilot study and face validity by experts. The pilot was done on 10 patients to validate the data which were discarded and were not included in the final analysis. The first section covered socio-demographic characteristics (age, sex, residence area, past medical history, and patient status: ambulatory and immobile). The second section comprised information pertinent to COVID-19 infection including results of the PCR and the signs and symptoms experienced by the patient. COVID-19 severity was assessed as per the World Health Organization (WHO) severity classification into critical, severe, and non-severe (World Health Organization, 2020). Critical COVID-19 was defined by the criteria for acute respiratory distress syndrome (ARDS), sepsis, septic shock, or other conditions such as mechanical ventilation (invasive or non-invasive) or vasopressor therapy. Severe COVID-19 was defined as having any of the following: oxygen saturation <90% on room air, respiratory rate >30 breaths/min in adults, and signs of severe respiratory distress (accessory muscle use, inability to complete full sentences). Non-severe COVID-19 was defined as the absence of any criteria for severe or critical COVID-19. The third section collected data related to laboratory values and coagulation parameters which comprised prothrombin time (PT), fibrinogen, D-dimer, platelet count, protein C, procalcitonin, International Normalised Ratio (INR), and blood oxygen saturation. The fourth section gathered all information related to the type of thrombosis development, anti-coagulants, and antiplatelets administered along with an assessment of hospitalisation status.

### 2.3. Sample size

This case–control study was conducted to determine the odds and the associated factors for thrombosis development in COVID-19 patients; however, there was no reference value to base the sample size calculations on. Therefore, it was decided to take all patients diagnosed with COVID-19 and then group them into patients who developed thrombosis as cases and patients who were thrombosis-free as control group.

### 2.4. Ethical approval

Ethics approval was obtained from the involved hospital sites, with a waiver of individual patient informed consent as it is a retrospective study that does not involve any direct patient contact.

### 2.5. Statistical analysis

Data were analysed using the SPSS software version 25. Counts/percentages and means/standard deviations were used to describe categorical and continuous variables, respectively. The Student *t*-test was used to compare two means. For categorical variables, the *χ*^2^ and Fisher exact tests were used (if the cell count is less than 5). Multivariable logistic regression model was used to assess the association between the development of thrombosis and the presence/absence of previous diseases, prior intake of medications, hospital floor that showed a *P *< 0.2 in the bivariate analysis (Bursac et al., 2008). Potential confounders variables were eliminated only if *P *> 0.2, to protect against residual confounding.

## 3. Results

### 3.1. Demographic data and comorbidities

**Figure 1.**
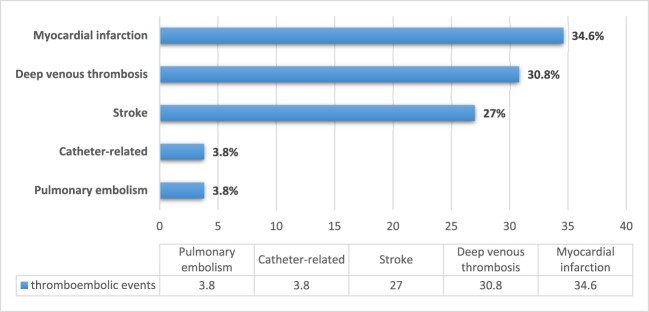
Types of thromboembolic events in hospitalised patients with COVID-19.

Among the 267 hospitalised COVID-19 patients included in our study, the mean age was 61.6 ± 15.4 years and 104 (39%) were females. Out of the total, acute thrombotic events occurred in 26 (9.7%); 9 patients had myocardial infarction (34.6%), 8 (30.8%) had deep vein thrombosis, 7 (27%) had ischemic stroke, 1 (3.8%) had pulmonary embolism, and 1 (3.8%) had catheter-related thrombosis (Figure 1).

The mean age of the patients who developed thrombotic episode was 62.58 ± 11.3 years and 80.8% were males. The demographic data and clinical characteristics of the cases and control groups are presented in Table 1.

**Table 1.** Demographic and clinical characteristics of COVID patients with and without thromboembolic events.

Variable	Total (*N* = 267)	Case (*N* = 26)	Control (*N* = 241)	*P*-value
*No. of patients, n (%)*	267	26 (9.7)	241 (90.3)	
*Age, y (mean ± SD)*	61.6 ± 15.4	62.6 ± 11.29	61.5 ± 15.8	0.730
*Age group, y*				
≤45	41 (15.4)	3 (11.5.3)	38 (15.8)	0.764
46–64	98 (36.7)	11 (42.3)	87 (36.1)	
≥65	128 (47.9)	12 (46.2)	116 (48.1)	
*Gender*				
Male	163 (61)	21 (80.8)	142 (58.9)	**0**.**03**
Female	104 (39)	5 (19.2)	99 (41.1)	
*BMI, (mean ± SD)*	28.5 ± 4.6	29.9 ± 7.1	28.3 ± 4.2	0.365
*BMI group, kg/m^2^*				
<25	26 (19.5)	2 (12.5)	24 (20.5)	0.737
≥25	107 (80.5)	14 (87.5)	93 (79.5)	
*Co-morbidities*				
Coronary artery disease	94 (35.2)	9 (34.6)	85 (35.3)	0.947
Diabetes mellitus	95 (35.6)	12 (46.2)	83 (34.4)	0.236
Hypertension	115 (43.1)	17 (65.4)	98 (40.7)	**0**.**016**
Dyslipidaemia	36 (13.5)	6 (23.1)	30 (12.4)	0.136
Chronic kidney disease	24 (9.1)	4 (15.4)	20 (8.3)	0.269
Peripheral artery disease	17 (6.5)	2 (7.7)	15 (6.2)	0.675
Previous thromboembolic event	4 (1.5)	2 (7.7)	2 (0.8)	**0**.**048**
*Medication history*				
Anti-platelet (aspirin)	85 (31.8)	12 (46.2)	73 (30.3)	0.099
Anti-hypertensive	96 (36)	16 (61.5)	80 (33.2)	**0**.**004**
Cholesterol-lowering	50 (18.7)	10 (38.5)	40 (16.6)	**0**.**014**
Anti-diabetics	91 (34.1)	11 (42.3)	80 (33.2)	0.352
*Patient status during hospitalisation*				
Ambulatory	231 (86.5)	24 (92.3)	207 (85.9)	0.548
Immobile (bedridden)	36 (13.5)	2 (7.7)	34 (14.1)	
*Disease severity*				
Critical COVID-19	87 (32.6)	16 (61.5)	71 (29.5)	**0**.**004**
Severe COVID-19	24 (9)	1 (3.8)	23 (9.5)	
Non-severe COVID-19	156 (58.4)	9 (34.6)	147 (61)	

*Data are presented as mean ± standard deviation for continuous variables and number (%) for categorical variables.

This study elected to use the WHO severity definitions based on clinical indicators, adapted from WHO COVID-19 disease severity categorisation.

Numbers in bold indicate significant *p*-values.

Our results showed that thromboembolic events were more significantly prevalent in males compared to females (80.8% vs 19.2%; *p* = 0.03). In terms of comorbidities and past medical history, no differences were identified between cases and controls except for hypertension (*p* = 0.016) and previous thromboembolic event (*p* = 0.048). As for the medication history, the use of anti-hypertensive drugs at home (*p* = 0.004) along with cholesterol-lowering drugs (*p* = 0.014) were shown to have a significant association with thrombosis occurrence during hospitalisation. Moreover, a significant association was found between disease severity (critical, severe, non-severe COVID-19) and development of thromboembolic events with a *p*-value of 0.004.

### 3.2. Bivariate analysis

#### 3.2.1. Laboratory data and abnormalities

Regarding the laboratory parameters considered in Table 2, thrombocytopenia was more significantly associated with cases compared to the control group (40% vs 14.5%) with a *p*-value of 0.003. No other associations were noted.

**Table 2.** Laboratory data of COVID-19 patients with and without thromboembolic events.

Variable	Case (*N* = 26)	Control (*N* = 241)	*P*-value
*D-dimer, ng/ml*	5932 ± 6927.1	3097 ± 3041.0	0.093
Normal	1 (5.3)	10 (4.9)	0.948
Elevated	18 (94.7)	193 (95.1)	
*CRP, mg/dl*	114.9 ± 131.1	35.6 ± 68.6	0.007
Normal	1 (3.8)	2 (0.9)	0.274
Elevated	25 (96.2)	230 (99.1)	
*Ferritin, ng/ml*	3527.3 ± 5837.2	1297.6 ± 2113.5	0.106
Normal	1 (5)	19 (9.2)	1.000
Elevated	15 (95)	188 (90.8)	
*Serum Creatinine, mg/dl*	2.6 ± 2.5	1.6 ± 1.5	0.052
Normal	12 (46.2)	156 (64.7)	0.062
Elevated	14 (53.8)	85 (35.3)	
*Haematocrit, %*	36.8 ± 8.2	35.5 ± 5.9	0.442
Normal	6 (24)	59 (24.7)	0.940
Low	19 (76)	180 (75.3)	
*Haemoglobin, g/dl*	11.9 ± 2.7	11.9 ± 2.4	0.951
Normal	10 (40)	99 (41.3)	0.904
Low	15 (60)	141 (58.7)	
*Platelets, k/µl*	188.7 ± 95.8	228.8 ± 95.6	0.047
Low	10 (40)	34 (14.5)	**0**.**003**
Normal	15 (60)	201 (85.5)	
*Oxygen saturation,%*	88.7 ± 9.8	89.5 ± 8.9	0.677
Normal	11 (44)	83 (36.6)	0.466
Low	14 (56)	144 (63.4)	

*Data are presented as mean ± standard deviation for continuous measures and number (%) for categorical variables.

Reference normal ranges for each parameter: D-dimer (<250 ng/ml), CRP (0-0.6 mg/dl), Ferritin (4.63–204 ng/ml), Cr (0.5–1.3 mg/dl), Haematocrit (Females: 37%–47%; Males: 42%–52%), Haemoglobin (Females: 12–16 g/dl; Males: 13.5–18 g/dl), Platelets (140–440 k/µl), oxygen saturation PO_2_ (≥93%).

Numbers in bold indicate significant *p*-values.

#### 3.2.2. Treatments, complications, and hospital status

During hospitalisation, the major complications included mechanical ventilation 81 (30.3%), acute respiratory distress syndrome 36 (13.7%), and bleeding events associated with the use of anticoagulants during hospitalisation 16 (6%) patients. Additionally, 109 (40.8%) patients were admitted to the ICU and 236 (88.4%) required supplemental oxygen therapy.

Fifty-four patients (20.2%) were on therapeutic dose of anticoagulants during hospital admission either because of the high clinical suspicion of thrombotic disease or due to thrombotic complications; 198 (74.2%) received only prophylactic dose anticoagulation and 15 (5.6%) did not receive any anticoagulation. As for the type of anticoagulant received, 112 (41.9%) were on heparin, 113 (42.3%) on low molecular weight heparin (LMWH), and 27 (10.1%) on direct oral anticoagulants (DOAC). Around 53.6% patients were discharged home and 32.6% patients passed away. Treatments, complications, and hospital status are shown in Table 3.

**Table 3.** Treatments, complications, and hospital status.

Variable	Total (*N* = 267)	Case (*N* = 26)	Control (*N* = 241)	*P*-value
*Complications*				
Oxygen therapy	236 (88.4)	20 (76.9)	216 (89.6)	0.097
Mechanical ventilation	81 (30.3)	16 (61.5)	65 (27)	**<0**.**001**
Acute respiratory distress syndrome	36 (13.7)	1 (5.3)	35 (14.3)	0.486
Bleeding event	16 (6)	7 (26.9)	9 (3.7)	**<0**.**001**
*Hospital floor*				
Intensive care unit (ICU)	109 (40.8)	19 (73.1)	90 (37.3)	**<0**.**001**
*Baseline anticoagulation*				
None	15 (5.6)	0	15 (6.2)	**<0**.**001**
Prophylactic anticoagulation	198 (74.2)	10 (38.5)	188 (78)	
Therapeutic anticoagulation	54 (20.2)	16 (61.5)	38 (15.8)	
*Type of anticoagulant*				
Low molecular weight heparin (LMWH)	113 (42.3)	15 (57.7)	98 (40.7)	0.122
Heparin	112 (41.9)	11 (42.3)	101 (41.9)	
Direct oral anticoagulants (DOAC)	27 (10.1)	0	27 (11.2)	
*In hospital anti-platelet*	140 (83.8)	19 (86.4)	121 (83.4)	1.000
*Length of hospital stay*	10.8 ± 8.2	11.2 ± 7.4	10.8 ± 8.3	0.826
*Clinical outcome*				
Discharged	143 (53.6)	2 (7.7)	141 (58.5)	**<0**.**001**
Still at hospitalised at the time of study conductance	37 (13.9)	6 (23.1)	31 (12.9)	
Inpatient death	87 (32.6)	18 (69.2)	69 (28.6)	

*Data are presented as mean ± standard deviation for continuous measures and number (%) for categorical measures.

Numbers in bold indicate significant *p*-values.

The use of mechanical ventilation (61.5% vs 27%, *p* < 0.001) and occurrence of bleeding associated with the use of anticoagulants (26.9% vs 3.7%, *p* < 0.001) were significantly higher in the cases compared to the controls. Also, ICU patients were significantly more likely to develop thromboembolic events than non-ICU patients (73.1% vs 37.3%, *p* < 0.001). In addition, a significant association was found between the use of anticoagulants at baseline and the cases (*p* < 0.001). Also, in-hospital death was significantly higher in cases compared to the control group (69.2% vs 28.6%, *p* < 0.001).

### 3.3. Multivariate analysis

When considering thromboembolic events development as the dependent variable, our analysis showed that the odds of thromboembolic event occurrence in hospitalised COVID-19 patients was significantly lower in females compared to males (aOR = 0.3), in those who received prophylactic anticoagulation during hospital admission (aOR = 0.1), and in patients classified as having non-severe COVID-19 (aOR = 0.3). The results of the multivariate model are shown in Table 4. On the other hand, having a history of a thromboembolic event (aOR = 9.2) and taking an antihypertensive medication at home (aOR = 3.1) were significantly associated with an increased odd of thrombosis. Also, our analysis revealed that cases had greater hospital stay than the control group (aOR = 0.1).

**Table 4.** Multivariate logistic regression models: risk factors associated with thromboembolic events development in hospitalised COVID-19 patients.

	B	*P*-value	Exp (B)	95% C.I. for Exp (B)	
			OR	Lower	Upper
Gender (females to males*)	−1.108	0.035	0.3	0.118	0.925
Anti-hypertensives (Yes to No*)	1.137	0.009	3.1	1.326	7.327
Prophylactic anticoagulation (Yes vs No*)	−2.069	<0.001	0.1	0.053	0.300
Disease severity (non-severe vs critical COVID-19*)	−1.298	0.004	0.3	0.114	0.656
Previous thromboembolic event (Yes vs No*)	2.215	0.040	9.2	1.108	75.707
Hospital discharge (Yes vs No*)	−2.565	0.001	0.1	0.017	0.352

*Variables entered*: gender, anti-platelet (aspirin) at home, cholesterol lowering medications at home, anti-hypertensive medications at home, type of anticoagulant used at hospital, oxygen therapy, mechanical ventilation, bleeding events, baseline anticoagulation upon hospital admission, hypertension, dyslipidaemia, previous thromboembolic event, COVID severity (critical, severe and non-severe COVID-19), ICU admission, clinical outcome (discharged, still at hospital, inpatient death).

*Reference group.

## 4. Discussion

This is the first study in Lebanon to assess the odds and associated factors for thrombosis development among COVID-19 patients. The main finding of this study is that the risk of thrombosis development in COVID-19 patients is higher in previous history of a thromboembolic event and previous intake of anti-hypertensives at home. However, females, intake of anticoagulants during hospital admission, and non-severe classification of COVID-19 were at lower risk of thrombosis development. Also, the study revealed that COVID-19 patients with thromboembolic events exhibited lower discharge rate compared to the control group.

### 4.1. Odds of thromboembolism

The cumulative frequency of thrombosis development among COVID-19 positive patients in our study was 9.7%; both venous and arterial types of thrombosis were observed with myocardial infarction, deep venous thrombosis and ischemic stroke being the most common types.

This is lower than the reported frequency of thrombosis development in previous literature which ranged from 18% to 36% (Helms et al., 2020; Pellegrini et al., 2021). Our findings can be explained by the limited diagnostic sensitivity of ultrasonography assessments, absence of uniform screening to exclude patients with previous history of thrombosis, and lack of systematic assessment with varying screening practices guided by clinicians’ suspicion, and varying follow-up time (Mohamed, 2021). The assessment of the rate of thrombosis development depends on other factors as daily activity mandated by home quarantine and alterations in medication adherence (Bikdeli et al., 2020).

### 4.2. Disease severity

Our study results showed that non-severe COVID-19 patients had lower risk of thrombosis development in line with a meta-analysis which supported the relation between COVID-19 severity along with changes in primary and secondary haemostatic parameters characterised by longer prothrombin time (PT), higher D-dimer values, and lower platelet count (Di Minno et al., 2020). In addition, severe COVID-19 is associated with multiple organ dysfunctions that leads to an increased macrovascular and microvascular thrombosis and triggers complication occurrences (Al-Ani et al., 2020; Rapkiewicz et al., 2020; Zhang et al., 2019).

#### 4.2.1. Impact on death

Our results showed that patients with thromboembolic events exhibited lower hospital discharge rate consistent with the results of another study (Zhang et al., 2020).

Our findings can be explained by the respiratory syndrome accompanied with severe disabling complications and the hypercoagulable state in COVID-19 patients that may lead to a high thrombotic risk with dramatic impact on mortality (Wichmann et al., 2020).

#### 4.2.2. Impact of anticoagulants

Our findings showed that the intake of anticoagulants during hospital admission was associated with a lower risk of thrombosis development consistent with the findings of another study (Tang, Bai, et al., 2020). It has been highlighted that the implementation of standardised thrombo-prophylaxis protocols in COVID-19 patients in the absence of contraindications mitigates the risk of developing thromboembolism and reduces the mortality risk associated with concomitant thrombosis and COVID-19 (Malas et al., 2020). Furthermore, several studies recommend the use of thromboprophylaxis in all hospitalised COVID-19 patients regardless of the disease severity classification since lower mortality and complications were encountered (American Society of Hematology, 2021; Bikdeli et al., 2020; Gerotziafas et al., 2020; Oudkerk et al., 2020; Thachil et al., 2020).

#### 4.2.3. Previous history of thromboembolic event and impact of aspirin intake

Our study findings highlighted that patients with previous history of thromboembolic event and anti-hypertensive intake were at higher risk of thrombosis development consistent with the findings of other studies (Kollias, Kyriakoulis, Dimakakos, et al., 2020; Kollias, Kyriakoulis, Stergiou, et al., 2020). According to a previous study, in addition to the COVID-19 infection itself, several comorbidities, such as hypertension, atrial fibrillation, heart failure, and peripheral artery disease, may contribute to thromboembolic risk even in mild or moderate COVID-19 patients (Li et al., 2021).

### 4.3. Limitations

There were several limitations in this study. First, the low odds of confirmed thromboembolism may reflect medical resource limitations in Lebanon especially during the COVID-19 outbreak and after the port blast. Second, we used electronic medical records in the present study which may be limited by incomplete or missing data. Furthermore, data about the previous history of factor five Leiden mutation is missing which is a well-known risk factor for thrombosis development and is highly common among Lebanese patients (Kreidy, 2012). In addition, our patient sample was from four sites only which might restrict the generalisability of our results to other patient populations. The sample size used in our analysis might have been small to generalise our findings to larger patient populations. Finally, residual confounding is possible since it is likely that we did not take all confounders of the association into account, particularly the ones related to endogenous hereditary thromboembolic diseases and the risk of polycythemia. Thus studies including the effect of inherited diseases are strongly needed to further refine the associations.

## 5. Conclusion

This study highlights that COVID patients are prone to develop thrombosis which is highly dependent on many associated risk factors such as diseases, previous medication intake, and laboratory parameters. Thus the study findings support the need to initiate active treatment with anti-thrombotic to manage thromboembolism, need of adequate screening procedures, and monitor the abnormal laboratory parameters. While waiting for data from a larger number of studies, the high thromboembolic risk of COVID-19 patients should be considered to timely predict thromboembolic risk, manage the long-term outcomes, and intervene with prophylactic measures to lower fatal complications.

## Data Availability

All data generated or analysed during this study are not publicly available to maintain the privacy of the individuals’ identities. The dataset supporting the conclusions is available upon request to the corresponding author.
